# Postoperative Pain Control by Intercostal Nerve Block After Augmentation Mammoplasty

**DOI:** 10.1007/s00266-017-0802-6

**Published:** 2017-08-08

**Authors:** Chang Min Kang, Woo Jeong Kim, Sean Hyuck Yoon, Chul Bum Cho, Jeong Su Shim

**Affiliations:** 1 Department of Plastic and Reconstructive Surgery, School of Medicine, Catholic University of Daegu, Duryugongwon-ro 17-gil, Nam-gu, Daegu, 705-718 Republic of Korea; 2 V-plastic Surgery, Daegu, Republic of Korea

**Keywords:** Augmentation mammaplasty, Pain management, Intercostal nerve block

## Abstract

**Background:**

In breast augmentation with implant, there is severe pain due to damage from expansion of breast tissue and the pectoralis major. Therefore, the authors conducted this study to analyze the effectiveness of postoperative intercostal nerve block (ICNB) in reducing postoperative pain after breast augmentation with implant.

**Method:**

Forty-four female patients were enrolled in the study. Just before awaking from general anesthesia, 34 cases were injected with 0.2% ropivacaine to both third, fourth, fifth, and sixth intercostal spaces. We compared them (ICNB group) with the control group for VAS scores at the time of arrival in the recovery room, after 30, 60, and 120 min.

**Result:**

The average VAS scores per time of the control group and ICNB group were 7.1 ± 0.74 and 3.50 ± 1.81 at arrival time in the recovery room, 7.00 ± 0.67 and 3.03 ± 1.47 after 30 min, 5.50 ± 0.71 and 2.68 ± 1.49 after 60 min, and 4.60 ± 0.84 and 2.00 ± 1.35 after 120 min. VAS scores of two groups were significantly different at each time and decreased overall. Also, time and group effect of the two groups were significantly different, especially between 30 and 60 min.

**Conclusion:**

ICNB just before awaking from general anesthesia showed a statistically significant reduction in VAS score, and this means postoperative pain was reduced effectively and time to discharge could be shortened. Therefore, it can be a good way to reduce postoperative pain after augmentation mammoplasty with implant.

**Level of Evidence IV:**

This journal requires that authors assign a level of evidence to each article. For a full description of these evidence-based medicine ratings, please refer to the Table of Contents or the online Instructions to Authors www.springer.com/00266.

**Electronic supplementary material:**

The online version of this article (doi:10.1007/s00266-017-0802-6) contains supplementary material, which is available to authorized users.

## Introduction

Augmentation mammoplasty using a breast prosthesis requires the insertion of an implant under breast tissue. Implant insertion levels are classified as subglandular, subpectorial or dual-plane based on the pectoralis major. Generally, subglandular insertion can be applied in patients with abundant breast tissue and mild glandular hypomastia, and in such cases soft breast tissue should be enough to cover the implant [1]. However, in Korean women, subpectoral or dual-plane insertion is mainly performed to reduce sagging after surgery and to avoid problems of implant palpability and visuality due to poor breast tissue after subglandular insertion.

Pain after subpectoral or dual-plane insertion is severe due to the expansion of breast tissue and the pectoralis major muscle and damage to separated tissues, and thus, pain control after surgery is an important consideration. When general anesthesia is unwanted or contraindicated, intercostal nerve block with local anesthesia has been reported to provide an effective alternative [2] and good pain control after surgery. Furthermore, intercostal nerve block has also been reported to decrease the incidence of chronic pain after thoracostomy [3]. Based on these reports, the authors undertook this study to determine whether intercostal nerve block was effective for reducing pain after augmentation mammoplasty.

## Methods

1. Patients and Preoperative Evaluations

This study was conducted using a single-center, prospective design. Of the patients who underwent augmentation mammoplasty from April 2014 to June 2016, a total of 44 female patients were enrolled in the present study. All patients underwent the procedure on both sides.

All augmentation mammoplasties were performed by one plastic surgeon, and all procedures of intercostal nerve block were performed by one anesthesiologist. All patients were provided written, informed consent. Patients were excluded if they had breast disease (e.g., breast cancer or a breast mass), a history of chest surgery for any reason, or were scheduled for reoperation due to complications of previous augmentation mammoplasty The 44 patients underwent surgery purely for cosmetic purposes.

All patients underwent careful history taking and were asked to provide demographic information, including age, body mass index (BMI), height, and weight before surgery. In addition, American Society of Anesthesiologists scores (ASA score) were determined prior to surgery.

2. Operation and Anesthesia procedures

All patients were administered propofol 1–1.5 mg/kg intravenously before surgery; general anesthesia was performed using a laryngeal mask airway. All implants were placed in the dual-plane pocket through an inframammary fold incision.

**Fig. 1:**
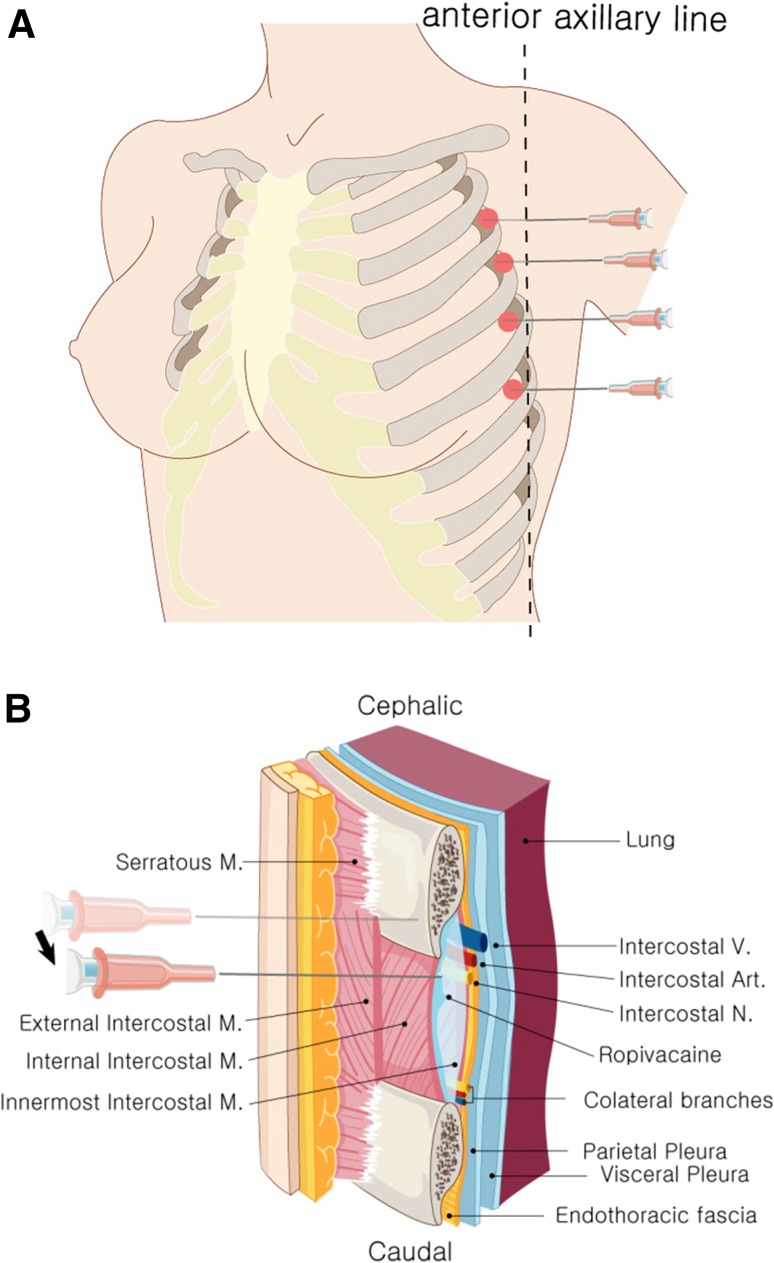
Illustrations of intercostal nerve block. **a** Injection sites for intercostal nerve block. Ropivacaine was injected at the point where both third, fourth, fifth, and sixth intercostal regions and both anterior axillary lines meet. **b** Anatomic diagram illustrating the relationship between the intercostal space and associated structure. The needle was walked off the inferior rib margin, while the clinician feels the sensation of touching the bone with the needle

After surgery was over and before waking from anesthesia, 34 subjects were administered an intercostal nerve block. With 15°–20° of cephalad angulation, a 23-gauge needle was advanced toward the inferior margin of the rib. The needle was then walked off the inferior rib margin while the clinician feels the sensation of touching the bone with needle and, at the end of the inferior rib margin, advanced 2–3 mm to lie adjacent to the intercostal nerve. After aspiration to check puncturing the plural or vessel, 2 ml of 0.2% ropivacaine was injected. This procedure was repeated at the point where both third, fourth, fifth, and sixth intercostal regions and both anterior axillary lines meet (Fig. 1) (Video 1). The other 10 patients awaked from general anesthesia without intercostal nerve block.

To compare pain levels after surgery, a 0–10 point visual analog scale (VAS) was used (0: no pain and 10: worst imaginable pain). VAS scores were checked for all 44 subjects on arrival in the recovery room and at 30, 60, and 120 min after arrival. In addition, the use of additional analgesics, discharge times, and complications were recorded.

Statistical analysis was performed using IBM SPSS, Version 19.0 (IBM Corp., Armonk, NY). In the study, variables about patient demographics, characteristics, operation and discharge data were summarized using mean ± standard deviation and to compare the ICNB and control groups about these variables, a two-sample *t* test was used. And variables about time course changes of VAS were compared using repeated measures two-factor analysis and multiple comparison by contrast. Statistical significance was accepted for *p* values <0.05.

## Results

The control (*n* = 10) and intercostal nerve block patients (ICNB group, *n* = 34) had average ages of 30.90 ± 7.37 and 31.50 ± 6.97, average heights of 162.20 ± 2.35 and 162.62 ± 3.95 cm, average weights of 50.20 ± 2.74 and 51.12 ± 4.82 kg, and average BMIs of 19.05 ± 0.66 and 19.04 ± 1.24 kg/m^2^, respectively (Table 1). Of the 44 study subjects, 43 were normal healthy patients without active disease (ASA score 1), and the other patient had an ASA score of 2 due to PVC bigeminy.

**Table 1:** Patient demographics and baseline characteristics

	ICNB	Control	*p* value^a^
Age	31.50 ± 6.97	30.90 ± 7.37	0.814
Height (cm)	162.62 ± 3.95	162.20 ± 2.35	0.753
Weight (kg)	51.12 ± 4.82	50.20 ± 2.74	0.570
BMI (kg/m^2^)	19.04 ± 1.24	19.05 ± 0.66	0.971

Results are presented as means ± SDs

*BMI* body mass index, *ASA* American society of anesthesiologists

^a^Result by two-sample *t* test

In the control and ICNB groups, average durations of surgery were 84.00 ± 15.06 and 85.44 ± 17.85 min, average durations of anesthesia were 111.00 ± 17.29 and 138.82 ± 24.19 min, and the average times from arrival at PACU (post-anesthesia care unit) to discharge were 489.00 ± 172.15 and 189.71 ± 15.17 min, respectively. The average sizes of implants used in the control and ICNB groups were 294.25 ± 20.28 and 296.99 ± 22.68 cc, respectively (Table 2).

**Table 2:** Operation and discharge data

	ICNB	Control	*p* value^a^
Operation time (min)	85.44 ± 17.85	84.00 ± 15.06	0.818
Anesthesia time (min)	138.82 ± 24.19	111.00 ± 17.29	0.002*
Implant size (cc)	269.99 ± 22.68	294.25 ± 20.28	0.701
Time to discharge (min)	189.71 ± 15.17	489.00 ± 172.15	0.000*

Results are presented as means ± SDs

*Statistically significant *p* values <0.05

^a^Result by two-sample *t* test

**Fig. 2:**
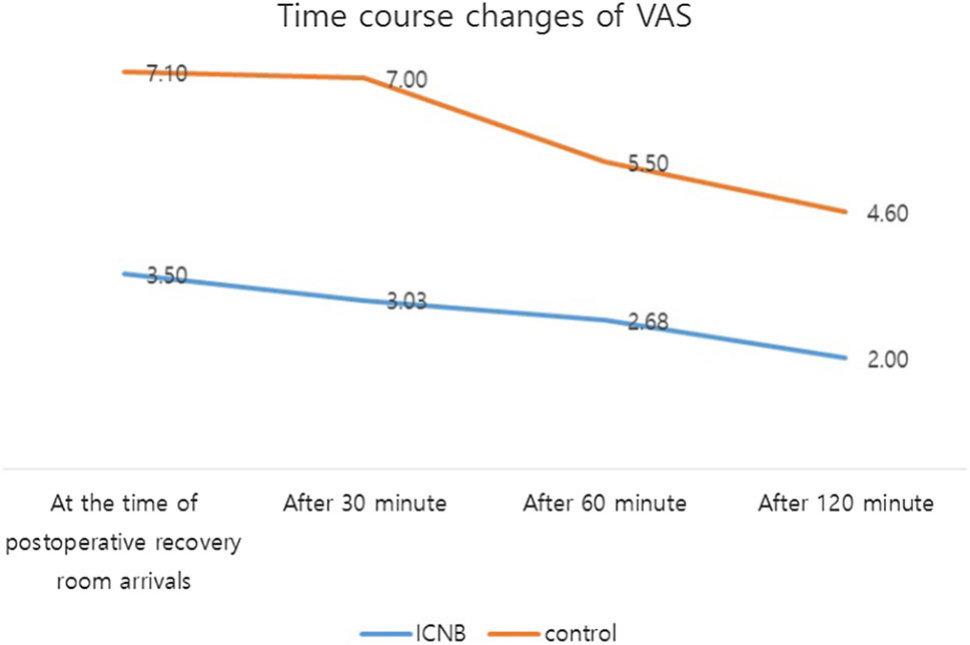
Time courses of VAS changes

In the control and ICNB groups, average VAS scores on arrival at the PACU, and 30, 60, and 120 min later were 7.10 ± 0.73 and 3.50 ± 1.81, 7.00 ± 0.66 and 3.03 ± 1.47, 5.50 ± 0.70 and 2.68 ± 1.49, and 4.60 ± 0.84 and 2.00 ± 1.35, respectively. At all four time points, VAS scores were significantly lower in the ICNB group (all *p* value = 0.000; Table 3; Fig. 2).

**Table 3:** Time course changes of VAS

Time course	ICNB	Control	*p* value^a^	*p* value^b^
At the time of postoperative recovery room arrivals	3.50 ± 1.81	7.10 ± 0.73	0.000*	T: 0.000* (1,2 > 3,4)^†^
After 30 min	3.03 ± 1.47	7.00 ± 0.66	0.000*	G: 0.000*
After 60 min	2.68 ± 1.49	5.50 ± 0.70	0.000*	T*G: 0.015* (1,2 > 3,4)^†^
After 120 min	2.00 ± 1.35	4.60 ± 0.84	0.000*	

Results are presented as means ± SDs

*VAS* visual analog scales

*Statistically significant *p* values <0.05

^a^Result by two-sample *t* test

^b^Result by repeated measures two-factor analysis (T: *p* value of time effect; G: *p* value of group effect, T*G: *p* value of interaction effect)

^†^Multiple comparison results by contrast

In both groups, time effect (T) and group effect (G) were statistically significant (T: 0.000, G: 0.000). In the multiple comparison result, regarding the time effect, no significant difference was observed between VAS scores at arrival and 30 min or between VAS scores at 60 and 120 min. However, differences between VAS scores at arrival, 30 and 60, 120 min were statistically significant.

In addition, significant differences were observed for time*group effects (T*G: 0.015). The difference between VAS scores at arrival and 30 min later, and the difference between the VAS scores at 60 and 120 min were not statistically significant, but the difference between VAS scores at arrival, 30 and 60, 120 min was statistically significant (Table 3).

No signs of complications, such as, hypotension, nausea, vomiting, injection site pain, systemic toxic reaction, abscess, neuritis, hematoma, and pneumothorax, were observed in any patient.

## Discussion

According to the statistics compiled by The International Society of Aesthetic Plastic Surgery (ISAPS) in 2015, augmentation mammoplasty is the third most common form of cosmetic surgery conducted worldwide. In Korea, augmentation mammoplasty is the fourth most common form of cosmetic surgery; over 20,000 surgeries are performed annually and numbers continue to increase [4]. Accordingly, there is an increasing demand to reduce postoperative pain and increase speed of recovery.

Recently, many studies have addressed postoperative pain after augmentation mammoplasty. The procedure is conducted by inserting implants underneath breast tissues, and thus, pain is believed to result from two causes. First, pain is produced by tissue injury during the dissection performed to place implants and by tissue swelling caused by implants. Since Korean women tend to have dense breasts with less breast tissue and fat, implants are usually inserted under the pectoralis major muscle to improve tactile satisfaction and prevent capsular contracture. However, during this procedure, the muscle can swell and cause severe pain. [5, 6] Second, pain may also be caused by postoperative complications, such as, inflammation or hematoma, caused by bleeding. Regardless of its cause, this pain requires hospitalization and inhibits rapid return to normal life.

Generally, analgesics are injected to reduce postoperative pain after augmentation mammoplasty, but their effects are not sufficient and it is unclear to what extent pain can be relieved or for how long. Pacik et al. [5, 6] compared postoperative pain after augmentation mammoplasty in patients that self-administered bupivacaine via indwelling catheters and patients that took systemic narcotics and found that the bupivacaine self-injection group exhibited more effective pain relief than the systemic narcotics group. Huang et al. [2] performed augmentation mammoplasty with intercostal nerve root block and local anesthesia using bupivacaine without general anesthesia and reported bupivacaine proved to be effective as an anesthetic agent and analgesic and that it also reduced hypertensive response and consequently prevented hematoma. Wolf and Clemens et al. [7] performed augmentation mammoplasty in 35 patients under general anesthesia with an additional paravertebral ropivacaine block at the T1–T6 level and found patients that received an additional paravertebral block had better pain control results.

In the present study, augmentation mammoplasty was performed in all 44 patients under general anesthesia, and postoperatively, intercostal nerves in breast areas were blocked just before awakening from general anesthesia. A significant intergroup difference in average anesthesia time was noted (111.00 ± 17.29 min in the control group and 138.82 ± 24.19 min in the ICNB group). However, the time required for one intercostal nerve block was about 10–15 s. And we repeated this procedure 8 times for one subject, so total time for the intercostal nerve block was 2–3 min. There were some subjects in the ICNB group who took more time to wake from general anesthesia, and it may be one reason for the time difference. But other factors could affect the difference, so further study might be needed to evaluate this difference.

We blocked bilateral third to sixth intercostal nerves using ropivacaine. Ropivacaine is effective for 640 ± 68 min at most and is almost as effective as bupivacaine, but its sensory block function is more sensitive [8]. And ropivacaine has been used for postoperative pain control [7, 9]. In the report of Coban et al. [9], effects of preoperative local ropivacaine infiltration on postoperative pain scores in infants and small children undergoing cleft palate repair were evaluated. In the study, the mean operation time was about 140 min, and the pain score at all observational postoperative periods was significantly lower in the ropivacaine group than in the control group.

Unlike cases in which intercostal nerve block is used to address anesthetic concerns, in the present study intercostal nerve block was administered for pain control, and thus, a VAS scale was used to assess pain. In the control and ICNB groups, average VAS scores were 7.10 ± 0.74 and 3.50 ± 1.81, 7.00 ± 0.67 and 3.03 ± 1.4, 5.50 ± 0.71 and 2.68 ± 1.49, and 4.60 ± 0.84 and 2.00 ± 1.35 after PACU arrival and at 30, 60, and 120 min after PACU arrival, respectively. Furthermore, at each time point, intergroup VAS score differences were significant. Furthermore, the time effect and the group effect were significant in both groups. However, in the ICNB group, no significant difference was observed between VAS scores at arrival and 30 min after arrival or between VAS scores at 60 and 120 min after arrival, but a significant difference was observed between VAS scores at arrival, after 30 min from arrival and after 60, 120 min from arrival. These findings indicate the efficacy of ropivacaine injected into intercostal tissues continued until discharge and that pain relief might be greatest during the period from 30 to 60 min after PACU arrival in the ICNB group.

Furthermore, average time to discharge was significantly shorter in the ICNB group than in the control group (189.71 ± 15.17 vs. 489.00 ± 172.15 min). One subject in the control group complained of postoperative pain, and she wanted to stay in the hospital to the next day (955 min). Because of the smaller number in the control group, this factor may have exaggerated the time to discharge of the control group. However, the average time to discharge excluding the subject was 437.22 min. Thus, intercostal nerve block was found not only to reduce pain effectively after surgery but also to reduce hospitalization times.

The VAS scores measured in the ICNB group of the present study were lower than those reported by Shim et al. [10], which were 7.7, 6.1, and 4.6 at 6, 24, and 48 h after surgery. However, these values were obtained in patients who underwent general anesthesia and subsequent ropivacaine administration immediately before surgical procedures were conducted. Thus, this difference is considered to be due to the fact that the maximum duration of action of ropivacaine is 640 ± 68 min, and thus, it is thought ropivacaine administration in this previous study more effectively blocked intercostal nerves after surgery and before awakening from anesthesia than blocking intercostal nerves before surgery. In addition, the control group in this previous study had mean VAS scores of 7.7, 6.1, and 4.6 at 6, 24, and 48 h after surgery. Therefore, the present study demonstrates the effectiveness on postoperative pain relief.

Complications after intercostal nerve block include pneumothorax, general toxic effects through intravascular infusion of local anesthetics, pain at the injection site, abscess formation and neuritis [11, 12]. The incidence of pneumothorax after intercostal nerve block has been reported from 0.073 to 19% [12–16]. But Moore et al. [17] reported that no therapeutic intervention was required for all the pneumothoraxes after intercostal nerve block in the study. But Holzer et al. [12] reported a case of severe pneumothorax after intercostal nerve block for extirpation of breast cancer. In the report, the patient had a chronic lung disease, and the author demonstrated that caution is needed in this procedure for patients with chronic lung disease. Cooter et al. [18] suggested the clinical indications for performing a chest radiograph after paravertebral nerve block include sudden hyperventilation, persistent cough, chest pain, shoulder pain, postoperative SpO2 < 92%, asymmetric respiration and diminished breath sounds on either side of chest.

Ropivacaine was reported as less cardiac toxic (reduced systolic function) and neurotoxic (visual and hearing disturbance, dysarthria, and paresthesia) than bupivacaine when administrated into an artery or vein [19–21]. However, in the study of Kopacz et al. [22], 0.25% ropivacaine (56 ml) produced peak plasma levels less than those considered toxic when used in bilateral intercostal blockade (T5–T11). And in our study, there was also no cardiac toxicity and neurotoxicity after intercostal nerve block with ropivacaine.

In the present study, every block was done by the same anesthetist (C. B. J.) and no complications, such as pneumothorax, systemic toxic reactions, infection or hematoma, were observed. The findings of this study suggest that when fully understanding the anatomical structure and monitoring the complications by a skilled nursing team, intercostal nerve block offers a safe, straightforward, and effective means of reducing pain after surgery.

The limitations of the present study are that VAS scores after hospital discharge were not assessed, so this study cannot evaluate the long-term effect on postoperative pain control of the intercostal nerve block, and the unbalanced size of the control and ICNB groups (*n* = 10 and 34, respectively) can decrease the test of power of this study.

## Conclusion

In the present study, intercostal nerve block, when conducted just before recovery from general anesthesia, significantly reduced pain in patients undergoing augmentation mammoplasty, as determined by VAS scores. This finding means postoperative pain reduction was effectively achieved, and as a result, rapid recovery and discharge were achieved. Therefore, the study indicates intercostal nerve block conducted just before recovery from general anesthesia should be considered an effective means of pain reduction and worthy of additional study.

## Electronic supplementary material

Below is the link to the electronic supplementary material.

Video 1 Procedure of intercostal nerve block (MP4 9822 kb)
